# The effects of a caffeine-containing beverage on muscle explosiveness during ballistic bench throws

**DOI:** 10.1186/1550-2783-9-S1-P15

**Published:** 2012-11-19

**Authors:** Emily Kammerer, Tyler Krings, Stephanie Wojton, Elizabeth Scheckel, Eric Kuklinski, Kelsey Jacobs, Carly Homan, Jenna Veldhuizen, Stephen Siegle, Amanda Wright, Meghan McCann, Dawn Anderson, Lonnie Lowery

**Affiliations:** 1 Department of Health, Exercise and Rehabilitative Sciences, Winona State University, Winona, MN 55987, USA

## Background

There is limited information available regarding the effects of caffeine-containing drinks on high intensity exercise performance. We hypothesized that Redline^®^ energy drink would significantly increase (p<0.05) muscle explosiveness in bench throws (BT) when compared to an identical placebo (PLB) in recreationally fit subjects (n=16).

## Methods

After a day of dietary control and caffeine abstinence, otherwise fasted subjects performed four individual ballistic bench throws under two conditions (Redline^®^, PLB), with trials being separated by 48-96 hours. The peak force (FOR), peak power (POW), peak velocity (VEL), peak displacement (DSP), and maximum rate of force development (RFD) of the Redline^®^ trial were compared to PLB.

## Results

Early results suggest a significant increase in FOR (Redline^®^ 329.6 ± 108.8 N vs. PLB 322.9 ± 107.1 N [p= 0.015]); POW (Redline^®^ 468 ± 177 W vs. PLB 446 ± 175 W[p= 0.001]); and VEL (Redline^®^ 1.82 ± 0.18 m/s vs. PLB 1.76 ± 0.19 m/s [p=0.0035]); and a trend in the data (p<0.10) for DSP (Redline^®^ 0.92 ± 0.08 m vs. PLB 0.90 ± .10 m [p= .0665]); and RFD (Redline^®^ 529 ± 262 N/s vs. PLB 493 ± 219 N/s [p=0.0685]).

## Conclusions

These preliminary data supported our hypothesis that muscle explosiveness in the bench throw would increase under the influence of Redline^®^ energy drink.

